# Temporal and Spatial Transcriptional Fingerprints by Antipsychotic or Propsychotic Drugs in Mouse Brain

**DOI:** 10.1371/journal.pone.0118510

**Published:** 2015-02-18

**Authors:** Kensuke Sakuma, Hidetoshi Komatsu, Minoru Maruyama, Sachiko Imaichi, Yugo Habata, Masaaki Mori

**Affiliations:** 1 Cardiovascular and Metabolic Drug Discovery Unit, Pharmaceutical Research Division, Takeda Pharmaceutical Company Limited, Fujisawa, Kanagawa, Japan; 2 Central Nervous System Drug Discovery Unit, Pharmaceutical Research Division, Takeda Pharmaceutical Company Limited, Fujisawa, Kanagawa, Japan; 3 Integrated Technology Research Laboratories, Pharmaceutical Research Division, Takeda Pharmaceutical Company Limited, Fujisawa, Kanagawa, Japan

## Abstract

Various types of antipsychotics have been developed for the treatment of schizophrenia since the accidental discovery of the antipsychotic activity of chlorpromazine. Although all clinically effective antipsychotic agents have common properties to interact with the dopamine D2 receptor (D2R) activation, their precise mechanisms of action remain elusive. Antipsychotics are well known to induce transcriptional changes of immediate early genes (IEGs), raising the possibility that gene expressions play an essential role to improve psychiatric symptoms. Here, we report that while different classes of antipsychotics have complex pharmacological profiles against D2R, they share common transcriptome fingerprint (TFP) profile of IEGs in the murine brain *in vivo* by quantitative real-time PCR (qPCR). Our data showed that various types of antipsychotics with a profound interaction of D2R including haloperidol (antagonist), olanzapine (antagonist), and aripiprazole (partial agonist) all share common spatial TFPs closely homologous to those of D2R antagonist sulpiride, and elicited greater transcriptional responses in the striatum than in the nucleus accumbens. Meanwhile, D2R agonist quinpirole and propsychotic NMDA antagonists such as MK-801 and phencyclidine (PCP) exhibited the contrasting TFP profiles. Clozapine and propsychotic drug methamphetamine (MAP) displayed peculiar TFPs that reflect their unique pharmacological property. Our results suggest that transcriptional responses are conserved across various types of antipsychotics clinically effective in positive symptoms of schizophrenia and also show that temporal and spatial TFPs may reflect the pharmacological features of the drugs. Thus, we propose that a TFP approach is beneficial to evaluate novel drug candidates for antipsychotic development.

## Introduction

Schizophrenia is chronic and devastating mental disorder that affects approximately 1% of the world population [1]. The symptoms are very complex and generally subdivided into three categories: positive symptoms (e.g., delusion, hallucination and thought disorder), negative symptoms (e.g., apathy, poor social functioning and emotional blunting), and cognitive deficits. Since schizophrenia is a unique human dysfunction, it is difficult to judge the similitude of animal models that attempt to replicate aspects of its behavioral abnormality [2]. Combination of these diverse genetic and environmental risk factors may turn this illness into complex brain disorder.

Existing antipsychotic drugs have been developed primarily targeting G_i/o_-coupled dopamine D2 receptor (D2R) blockade based on dopamine hypothesis [3]. They are effective only for positive symptoms that are mainly due to mesolimbic dopamine hyperactivity in dopamine neurons. Negative symptom and cognitive impairment poorly respond to current medications and need better treatment. Antipsychotics can be commonly divided into two classes, the typical or first-generation antipsychotics and the atypical or second-generation antipsychotics. Typical antipsychotics such as haloperidol mainly possess an antagonistic activity for the D2R, whereas atypical antipsychotics such as olanzapine and clozapine show antagonistic activities for serotonin 2A (5-HT2A) receptor, D2R and other multiple G-protein coupled receptors (GPCRs). In particular, clozapine has a unique property distinguished from other antipsychotic drugs by virtue of its higher affinities for D1 dopamine receptor (D1R) and 5-HT2A receptor and its lower affinity for D2R and thus needs higher clinical dose, indicating that clozapine may not be primarily acting at the D2R [3, 4]. A new class of an antipsychotic drug, aripiprazole has affinity for various receptors but somewhat differs from other SDA-type (SDA; serotonin-dopamine antagonist) atypical drugs because it has the highest affinity for D2R receptor [5].

For two decades, compelling converging evidence indicates that modulating glutamatergic signaling may improve not only positive but also negative and cognitive symptoms (“glutamate hypothesis”) as another therapeutic strategy. Results of clinical studies conducted so far are promising but yet to be accomplished [6]. Beyond the dopamine receptor and glutamate receptor, a number of potential therapeutic targets have been identified and evaluated for schizophrenia drugs so far [7]. Included are various types of targets such as GPCRs and ion channels. Neuronal activity at these diverse target triggers downstream gene expression programs, leading to long-lasting synaptic modification and plasticity [8].

A new paradigm has emerged that depicting gene expression profile gives us translational bridge among cellular activity, rodent models and human patients [9, 10]. Transcriptome fingerprint (TFP) is the name of such a novel approach to distinguish hallucinogenic and non-hallucinogenic drugs by displaying their transcriptional patterns [9, 11]. Neuronal immediate early genes (IEGs) are genes whose rapid and transient response to various extracellular stimuli are earliest and their transcription have been implicated in neuronal plasticity and cognitive function [12, 13]. Mapping those transcripts by González-Maeso *et al* [9, 11] is assumed to be one powerful method for visualizing various brain disorders including schizophrenia. Indeed, multiple IEGs have been identified as susceptibility genes in human genetic screens for schizophrenia [14] or genes linked with schizophrenic signaling and antipsychotic process [15–17]. Among them, *c-fos* and *Arc* are the most familiar IEGs whose molecular and biological functions have been uncovered extensively during the past few decades [18–21]. In addition, members of EGR family have been identified as calcineurin-related susceptibility genes for schizophrenia, indicating that alteration in calcineurin-EGRs signaling downstream of both dopaminergic and glutamatergic cascades may cause schizophrenia pathogenesis [14]. The gene expression of *Sgk1*, serum/glucocorticoid-induced protein kinase-1, is regulated by atypical antipsychotic clozapine [16], while *Ccn1*, CCN family member 1 (also known as cysteine-rich angiogenic inducer 61 (CYR61)) is transcriptionally induced by propsychotics including MAP and PCP [17].

Antipsychotic drugs have been shown to induce IEG expressions in rodent brain regions that are associated with schizophrenia, which may be directly linked to their immediate therapeutic benefit [16, 22]. In this study, we investigated the effects of various classes of antipsychotics and propsychotics on IEG expressions in mouse brain after their acute administration. For this analysis, we selected *c-fos*, *Arc*, *Egr1*, *Egr2*, *Egr3*, *Sgk1* and *Ccn1* as IEGs on the basis of previously related reports and biological functions associated with psychiatric disorders as mentioned above. These TFP analyses provide us not only new insight into psychotic features at transcriptional level but also a novel *in vivo* system for predicting the potential antipsychotic activity of new compounds.

## Materials and Methods

### Animals

All experiments were performed using ICR and C57BL/6J mice purchased from Clea Japan, Inc. Animals were housed in an environment with 12 h dark-light cycle with light on at 7:00 a.m. and provided food (CE-2; Clea Japan, Inc.) and tap water *ad libitum*. Animals were allowed to acclimatize appropriately to the room before starting experiments. All experiments were reviewed and approved by the Laboratory Animals Ethics Committee of Takeda Pharmaceutical Company Limited.

### Preparation of Mouse Brain Samples

For IEGs expression analysis, ICR mice were used unless otherwise noted and habituated appropriately (as described above). Only for MAP and PCP dose-response experiments, C57BL/6J mice were used for experiments. Drugs were administered orally (p.o., suspended in 0.5% methylcellulose (Shin-Etsu Chemical)), intraperitoneally (i.p., dissolved in saline, sterile 0.9% NaCl (Otsuka Pharmaceutical Factory)) and subcutaneously (s.c., dissolved in saline same as the case of i.p.). At each time point, the mice were euthanized by decapitation and whole brains were collected. Fitting the brain on a Precision Brain Slicer (Braintree Scientific), four brain regions including prefrontal cortex, nucleus accumbens, striatum, and hippocampus were dissected on ice. Schematic representation of those brain regions is shown in S1 Fig. Here, prefrontal cortex was defined as the anterior part of the frontal lobe of the brain 1 mm behind the olfactory bulb. Nucleus accumbens, striatum, and hippocampus were dissected from both hemispheres with a 1 mm slice using a Precision Brain Slicer.

### RNA Preparation, Reverse Transcription and Real-Time Polymerase Chain Reaction (PCR)

Total RNAs from four brain regions were extracted using Isogen (Nippon Gene) according to the manufacturer’s instructions. Purified RNA was treated with the DNase I Amp Grade (Life Technologies) according to manufacturer’s instructions. The reverse transcription (RT) reactions were performed at 42°C with each RNA preparation using a random primer (Life Technologies) and SuperScript II reverse transcriptase (Life Technologies) according to the manual attached. After RT reactions were completed, the mixture was dissolved in TE buffer and the resulting cDNAs were used as templates for real-time PCR using a Prism 7900HT sequence detector (Applied Biosystems). Thermal cycling parameters were 2 min at 50°C, 10 min at 95°C, followed by 40 cycles of 95°C for 15 sec and 60°C for 1 min. The threshold cycle (Ct) values were converted to relative copy numbers using the oligomers listed in S1 Table as templates. All data were normalized by the house keeping gene *cyclophilin A*.

### Data Analysis and Statistics

Data analysis was performed with Prism 5 software (GraphPad Software). In analysis of dose-response studies, data were tested by Bartlett’s test for homogeneity of variance. When the variances were homogeneous, Williams’ test was performed. When the variances were heterogeneous, the Shirley-Williams’ test was performed. Bartlett’s test was conducted at the significance level of less than 0.05%, and the Williams’ and Shirley-Williams’ tests were conducted at the one-tailed significance level of less than 0.025%. In analysis of time-course studies, unpaired t-test with Welch’s correction was employed to compare the statistical significance between the drug administration and its corresponding vehicle group at each time point [23] and p<0.05 was considered statistically significant, as previously described by González-Maeso *et al* [9].

## Results

### TFPs of IEGs Following Acute Treatment with Typical and Atypical Antipsychotics in Mouse Brain Regions

**Fig 1:**
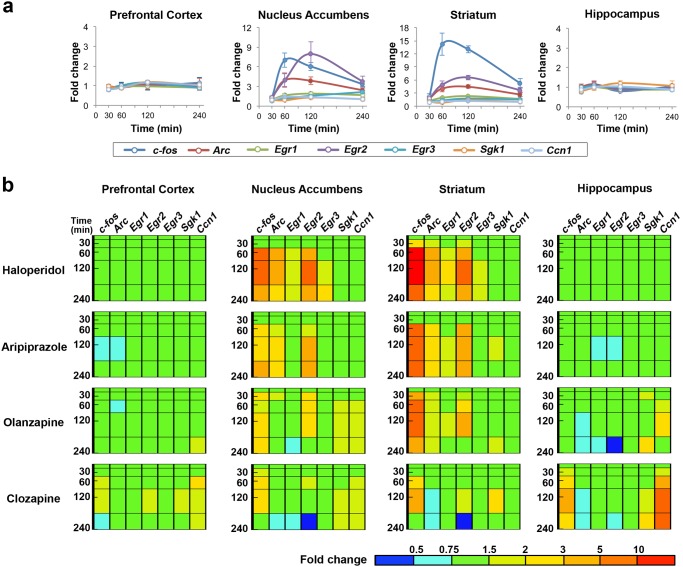
Antipsychotics induce distinctive IEG expression changes in nucleus accumbens and striatum, mainly due to their prominent D2R antagonistic activity. (a) Time courses of seven IEG expressions induced by 0.3 mg/kg (p.o.) of haloperidol. Data are shown as mean± SEM (n = 5). Filled circles, p<0.05 in unpaired t-test with Welch’s correction, compound (n = 5) versus vehicle (n = 5) at the same time point; open circles, not significant. The changes of expression levels are shown as fold change over vehicle. (b) TFP of seven IEGs over time induced by antipsychotic agents in mouse brain. Antipsychotics; haloperidol (0.3 mg/kg, p.o.), aripiprazole (3 mg/kg, p.o.), olanzapine (10 mg/kg, p.o.) and clozapine (30 mg/kg, p.o.). The bar depicted in the bottom right corner represents the expression level of the gene searched in a TFP. Red represents high expression, green represents vehicle-control level expression, and blue represents low expression.

We investigated whether TFPs in mouse brain could discriminate among different classes of antipsychotics *in vivo*. We first analyzed the time course of the TFPs of *c-fos*, *Arc*, *Egr1*, *Egr2*, *Egr3*, *Sgk1* and *Ccn1* by quantitative real-time PCR (qPCR) following acute administrations of typical antipsychotic haloperidol (0.3 mg/kg, p.o.), atypical antipsychotics olanzapine (10 mg/kg, p.o.) and clozapine (30 mg/kg, p.o.), and aripiprazole (3 mg/kg, p.o.) based on their effective behavioral doses in mice [24, 25] (Fig. 1). Mice were administered by these agents with treatment of 30, 60, 120, and 240 min. In mouse brain, prefrontal cortex, nucleus accumbens, striatum, and hippocampus were analyzed based on association with schizophrenic symptom or side effect profile of drug treatment [16, 22]. We found that all the tested antipsychotics significantly induced expression changes of the IEGs at ~60 min after their administration (Fig. 1 and S2 Table). Notably, haloperidol and aripiprazole showed indistinguishable TFPs even though they are quite different each other in the pharmacological profiles against D2R. Both of them commonly evoked robust gene expressions of *c-fos*, *Arc*, and *Egr2* in nucleus accumbens and striatum at peak point of 60 or 120 min (Fig. 1 and S2 Table). Similarly, olanzapine obviously induced *c-fos* and *Egr2* in nucleus accumbens, and *c-fos*, *Arc*, *Egr1* and *Egr*2 in striatum and *Ccn1* in hippocampus at peak point of 60 or 120 min (Fig. 1b and S2 Table). In prefrontal cortex, olanzapine-induced gene expressions were not changed more dramatically than in nucleus accumbens and striatum (Fig. 1b and S2 Table). In contrast, clozapine evoked unique TFPs at peak point of 120 min that differed from haloperidol, aripiprazole, and olanzapine. Clozapine affected the gene expressions in four regions and increased the transcripts of *c-fos*, *Sgk1* and *Ccn1* in almost all the regions (Fig. 1b and S2 Table).

**Fig 2:**
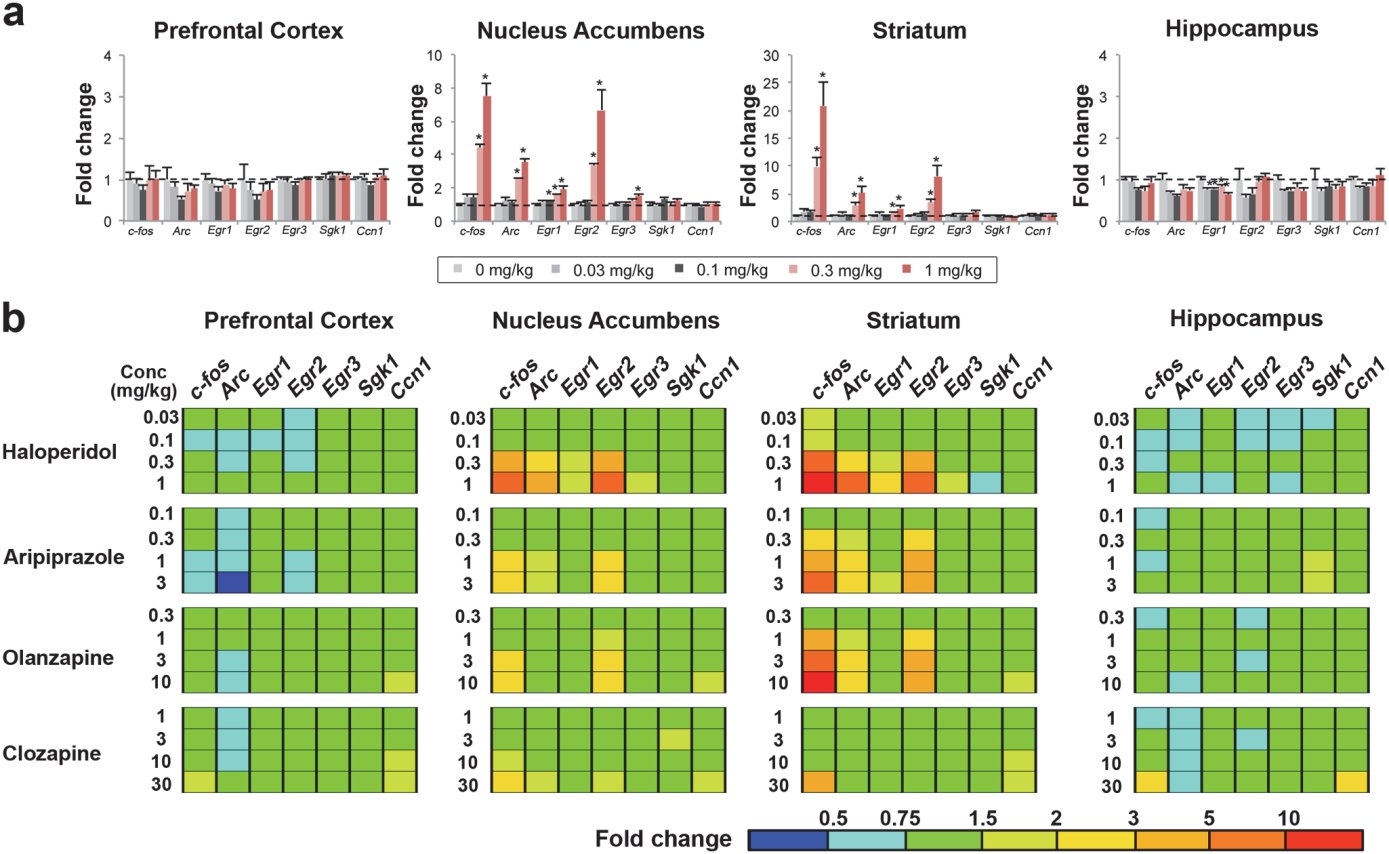
Antipsychotics treatment except clozapine commonly induce three IEGs, *c-fos*, *Arc* and *Egr2* in a dose-dependent manner in nucleus accumbens and striatum. (a) Dose-dependent effects of haloperidol (1 hr treatment, 0.03 mg/kg to 1 mg/kg, p.o.). Note that four IEGs (*c-fos*, *Arc*, *Egr1* and *Egr2*) are dose-dependently elevated in nucleus accumbens and striatum. The dashed horizontal line indicates baseline (the value of 1).Data are shown as mean± SEM (n = 4~5). *p<0.025, significantly different from vehicle at a dose of 0 mg/kg in Williams’ test. (b) TFPs of seven IEGs showing dose-response pattern by treatment of four antipsychotics in mouse brain. Significant induction for three IEGs (*c-fos*, *Arc* and *Egr2*) were commonly observed in nucleus accumbens and striatum among three antipsychotics, haloperidol, aripiprazole and olanzapine. Antipsychotics; haloperidol (1 hr treatment, 0.03 mg/kg to 1 mg/kg, p.o.), aripiprazole (2 hrs treatment, 0.1 mg/kg to 3 mg/kg, p.o.), olanzapine (1 hr treatment, 0.3 mg/kg to 10 mg/kg, p.o.) and clozapine (1 hr treatment, 1 mg/kg to 30 mg/kg, p.o.). The bar depicted in the bottom right corner represents the expression level of the gene searched in a TFP. Red represents high expression, green represents vehicle-control level expression, and blue represents low expression.

We next examined the dose-response relationship of IEG mRNA changes by antipsychotics. According to data in Fig. 1, the time point showing maximal transcript changes for each antipsychotic drug was selected for subsequent studies. The gene expression changes were analyzed at 60 min for haloperidol, olanzapine, and clozapine, while at 120 min for aripiprazole. Haloperidol (0.03 to 1 mg/kg, p.o.), aripiprazole (0.1 to 3 mg/kg, p.o.), olanzapine (0.3 to 10 mg/kg, p.o.), and clozapine (1 to 30 mg/kg, p.o.) evoked mRNA changes at 0.3, 0.3, 1, and 30 mg/kg, respectively (Fig. 2 and S3 Table). In the dose-response relationship, haloperidol, aripiprazole, and olanzapine mainly affected nucleus accumbens and striatum with homologous TFPs, and markedly increased the transcripts of *c-fos*, *Arc*, and *Egr2* (Fig. 2b and S3 Table). As seen in Fig. 2b, clozapine was also distinguished from other antipsychotics in the dose-response TFPs.

### TFPs of IEGs by Administration of D2R Agonist Quinpirole and Antagonist Sulpiride

**Fig 3:**
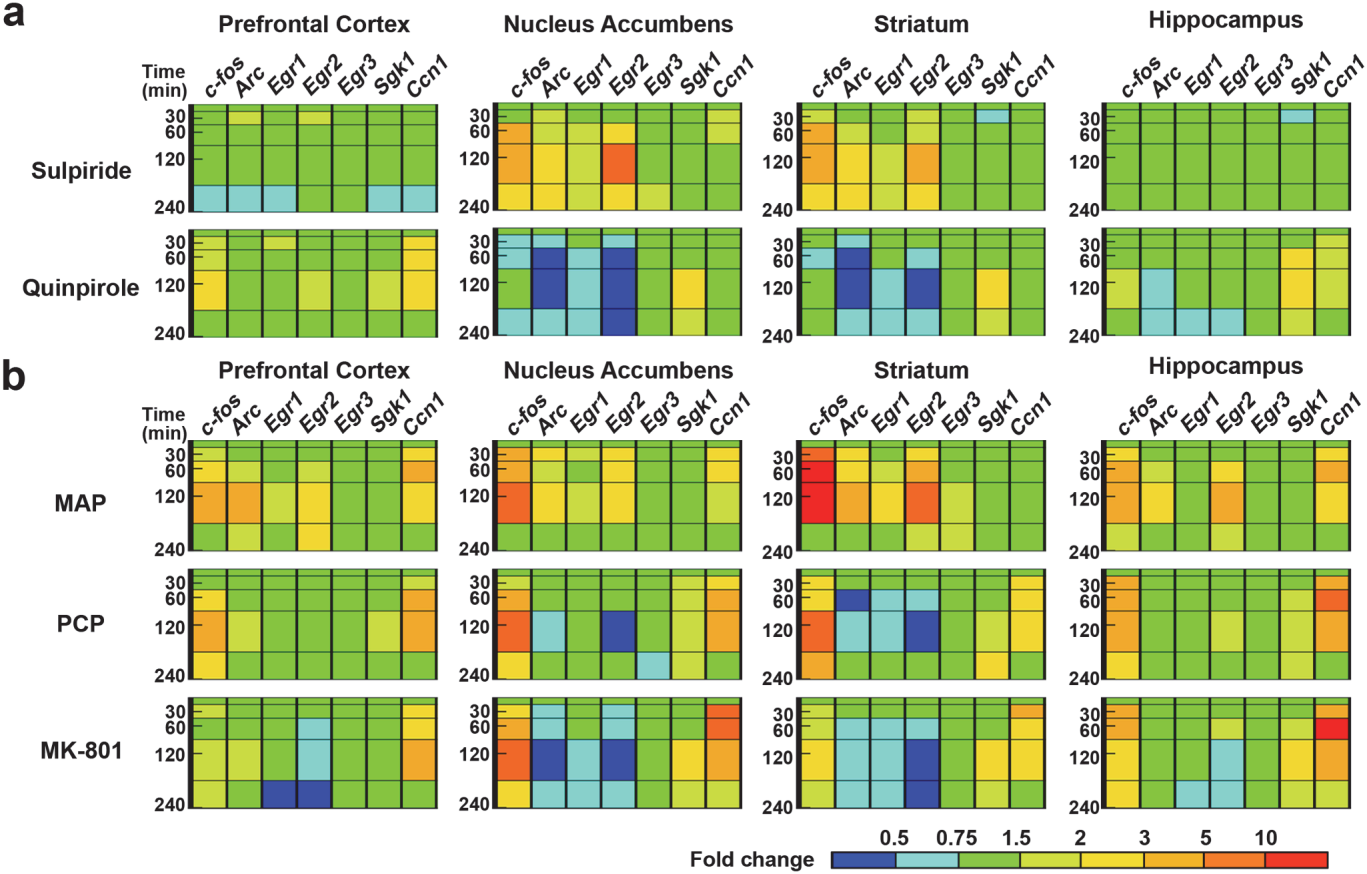
D2R antagonist shows spatial TFPs homologous to those by antipsychotics, while D2 agonist and propsychotic agents exhibit their peculiar TFPs. TFPs of seven IEGs over time induced by D2R antagonist/agonist and propsychotic drugs in mouse brain; (a) Selective D2R antagonist sulpiride (100 mg/kg, i.p.) and agonist quinpirole (10 mg/kg, i.p.), and (b) Propsychotic agents MAP (3 mg/kg, s.c.), PCP (10 mg/kg, s.c.) and MK-801 (1 mg/kg, s.c.). The bar depicted in the bottom right corner represents the expression level of the gene searched in a TFP. Red represents high expression, green represents vehicle-control level expression, and blue represents low expression.

Since it is well known that three antipsychotics which have shown the homologous TFPs in Fig. 1 share the high intrinsic activity on D2R, we next examined the time-course TFPs by D2R agonist quinpirole and antagonist sulpiride (Fig. 3a). Sulpiride (100 mg/kg, i.p.) primarily affected the transcripts in nucleus accumbens and striatum, and markedly increased the expressions of *c-fos*, *Arc* and *Egr2* at ~60 min. This TFP was closely homologous to that of haloperidol and aripiprazole. Meanwhile, quinpirole (10 mg/kg, i.p.) affected the mRNAs in four regions and significantly decreased the transcripts of *c-fos*, *Arc*, *Egr1* and *Egr2* in nucleus accumbens and striatum, and increased *Sgk1* mRNA in four regions and *Ccn1* mRNA in prefrontal cortex and hippocampus (Fig. 3a and S4 Table). In nucleus accumbens and striatum, quinpirole showed the contrasting TFP in comparison with that of sulpiride, haloperidol, and aripiprazole.

### TFPs of IEGs by Administration of Propsychotics, Methamphetamine and NMDA Antagonists, Phencyclidine and MK-801

**Fig 4:**
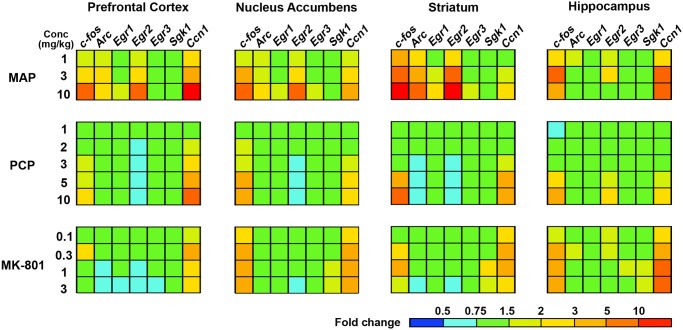
TFPs of seven IEGs by treatment of propsychotic agents showing dose-response pattern in mouse brain. Significant reduction of *Egr2* in nucleus accumbens, striatum and prefrontal cortex, and increases of *c-fos* and *Ccn1* in all tested regions are characteristic of NMDA antagonists, whilst MAP serves robust IEGs induction in all four regions studied here. The bar depicted in the bottom right corner represents the expression level of the gene searched in a TFP. Red represents high expression, green represents vehicle-control level expression, and blue represents low expression. Propsychotic agents; MAP (1 hr treatment, 1 mg/kg to 10 mg/kg, s.c.), PCP (1 hr treatment, 1 mg/kg to 10 mg/kg, s.c.), MK-801 (1hr treatment, 0.1 mg/kg to 3 mg/kg, s.c.).

A psychostimulant methamphetamine (MAP) causes excessive release of dopamine in brain and is thus used to mimic positive symptoms of schizophrenia, using animals [26]. MAP (3.0 mg/kg, s.c.) influenced the transcripts in four regions and conspicuously elevated *c-fos*, *Arc*, *Egr2* and *Ccn1* mRNAs at ~60 min (Fig. 3b and S5 Table). At 240 min, these gene expression changes mostly returned to baseline. In the dose-response relationship at 60 min, MAP (1 to 10 mg/kg, s.c.) also affected the gene expressions in four regions and dose-dependently increased *c-fos*, *Arc*, *Egr2* and *Ccn1* mRNAs from 1 or 3 mg/kg (Fig. 4 and S6 Table).

N-methyl-D-aspartate (NMDA) receptor antagonists such as phencyclidine (PCP) and MK-801 induce symptoms similar to those of schizophrenia in healthy patients and cause a resurgence of symptoms in stable patients [27]. Thus, an NMDA antagonist is used to mimic psychosis of schizophrenia. Both PCP (10 mg/kg, s.c.) and MK-801 (1 mg/kg, s.c.) affected the gene expressions in four regions and significantly down-regulated at ~60 or 120 min the expressions of *Arc* and *Egr2* in nucleus accumbens and striatum, *Egr1* in striatum, and increased *c-fos* and *Ccn1* mRNAs in every region (Fig. 3b and S5 Table). MK-801 also decreased *Egr2* mRNA level in prefrontal cortex and hippocampus (Fig. 3b and S5 Table). In the dose-response relationship at 60 min, PCP (1 to 10 mg/kg, s.c.) and MK-801 (0.1 to 3 mg/kg, s.c.) dose-dependently up-regulated *c-fos* and *Ccn1* mRNAs in almost every region, and reduced the transcripts of *Egr2* in three regions except hippocampus, and *Arc* in striatum (Fig. 4 and S6 Table). Likewise, MK-801 (0.1 to 3 mg/kg, s.c.) at 60 min also decreased *Arc* and *Egr1* mRNA in prefrontal cortex (Fig. 4 and S6 Table).

## Discussion

Among seven IEGs investigated herein, *c-fos* is well known as a nonspecific marker of neuronal activity [9]. EGR family and *Arc* have been recognized as components downstream of the NMDA receptor and demonstrated their role in synaptic plasticity processes. *Arc*, considered an index of neuronal activity, is selectively localized on dendritic spines and synapses where it associates with actin cytoskeleton and is suggested to be involved in both maintenance and consolidation of long-term memory [20, 28, 29]. EGR genes encode transcription factors with a highly conserved DNA binding domain consisting of three zinc finger motifs. *Arc* and *Egr2* are computationally predicted to have binding sites in their promoter regions for EGR family and CREB, respectively [30]. It has been reported that their expression levels are induced by CREB via cAMP/PKA signaling, leading to induction of long-term potentiation (LTP) through the activation of NMDA receptor [31]. EGR genes, which are schizophrenic susceptibility candidates and down-regulated at a transcript level in the prefrontal cortex of schizophrenic patients, are downstream targets for calcineurin that uniquely integrates dopaminergic and glutamatergic pathways and their altered function can contribute to pathogenesis of schizophrenia through various mechanisms such as neuronal development and plasticity [14, 32]. EGR genes also interact with a number of factors implicated in schizophrenia. For instance, *neuregulin 1* (*Nrg1*) has been identified as a susceptibility gene for schizophrenia [33]. All *Egr1*, *Egr2* and *Egr3* are downstream targets for *Nrg1* [34]. *Sgk1* is also assumed to be involved in memory consolidation and strikingly up-regulated by clozapine in rat brain [16, 35]. Taken all together, we strongly believe that these IEGs investigated herein are tightly linked to schizophrenia.

Our data show that antipsychotics clinically effective in positive symptoms of schizophrenia, haloperidol, aripiprazole, and olanzapine have homologous temporal and spatial TFPs showing a transcriptional induction of *c-fos*, *Arc*, and *Egr2*, particularly in nucleus accumbens and striatum (Figs. 1 and 2). Especially, the TFPs by haloperidol and aripiprazole closely resemble each other. Consistently, despite their distinct pharmacological properties, the meta-analysis demonstrated that typical and atypical antipsychotics as well as aripiprazole do not differ obviously in overall symptoms including positive and negative symptoms [36].

Nucleus accumbens and striatum, where D1R and D2R are most abundant [3, 37], have been well known to play a pivotal role for dopaminergic transmission from the ventral tegmental area (VTA) and the substantia nigra pars compacta [38]. Nucleus accumbens is a common site of therapeutic action of antipsychotic drugs due to the presence of D2R in this region and has been thought to be responsible for positive symptoms [39]. In accordance, haloperidol, aripiprazole, and olanzapine robustly induced the transcription of *c-fos*, *Arc*, and *Egr2* (Figs. 1 and 2), suggesting that the induction of these genes may be involved in improvement of positive symptoms of schizophrenia. These three antipsychotics affect gene expression in striatum more robustly than in nucleus accumbens while their TFPs in striatum and nucleus accumbens are almost identical. The striatum is a key region involved in motor coordination and is associated with antipsychotic drug-induced extrapyramidal symptom (EPS) [40]. Similar to nucleus accumbens, the striatum has a higher D2R density and is implicated in the original dopamine hypothesis of schizophrenia [39]. Thus, our data implicates that the induction of *c-fos*, *Arc*, and *Egr2* in nucleus accumbens and striatum by haloperidol, aripiprazole, and olanzapine are mediated through D2R in the dependence of its receptor expression level. In agreement with this, the D2R antagonist sulpiride induced these three gene expressions whereas the D2R agonist quinpirole conversely reduced them (Fig. 3a). More intriguingly, such characteristic TFPs induced by quinpirole showed high similarity with those by NMDA antagonists, PCP and MK-801 (Figs. 3a and 4), consistent with previous findings that NMDA currents can be abrogated by D2R activation [41, 42]. This suggests that D2R antagonism could lead to an induction of *c-fos*, *Arc*, and *Egr2* mRNAs by potentiating NMDA signaling in the basal ganglia. In this case, aripiprazole may also function as an antagonist because it can behave as a D2R antagonist dependent on the concentration of extracellular dopamine or in β-arrestin signaling pathway [5, 43, 44].

Several lines of evidence suggest that a dysfunction of glutamatergic neurotransmission contributes to the pathophysiology of schizophrenia. Included in this evidence is the fact that NMDA receptor antagonists such as PCP and MK-801 have schizophrenia-like effects in normal individuals and exacerbate pre-existing symptoms in schizophrenic patients [45]. Currently, the psychosis induced by NMDA antagonists derived considerable scientific interest since it represents possibly the most accurate pharmacological model of schizophrenia available [46]. Our data has shown that MK-801 significantly down-regulates the transcripts of *Arc*, *Egr1*, and *Egr2* in nucleus accumbens and striatum (Fig. 3b). This gene expression profile is nearly identical to that by PCP (Fig. 3b) whereas it shows quite opposite changes of the transcripts in comparison with that elicited by haloperidol, aripiprazole, and olanzapine which elevated the transcription of *Arc*, *Egr1*, and *Egr2* in striatum and nucleus accumbens (Figs. 1 and 2). NMDA hypofunction might generate dysregulation of dopamine systems that, in turn, further weakens NMDA-mediated connectivity and plasticity [47]. Our results suggest that PCP and MK-801 possess common features of cellular signaling that are translated into the unique transcriptome fingerprints, and antipsychotic agents may activate at least a part of those NMDA signal transduction. Further studies should be required whether various types of antipsychotics can restore the NMDA antagonist-mediated TFPs.

Clozapine improves not only positive symptoms but also negative symptoms and is well known as “magic shotgun” type drug acting on multiple receptors [48]. Clozapine exhibited a distinctive pattern of *c-fos*, *Sgk1* and *Ccn1* up-regulation in almost all four investigated regions including prefrontal cortex and hippocampus (Figs. 1 and 2). Our result implies the possibility that transcriptional mechanism of clozapine for improving various symptoms of schizophrenia is totally different from other antipsychotics. In addition, it is intriguing that high concentration of clozapine displayed the similar TFPs with those of NMDA antagonists (Figs. 1 and 3). Although the reason is unclear, those distinctive TFPs might reflect the unique therapeutic and adverse effects of clozapine.

MAP is well known to mimic positive symptoms in human [26], and here elicits induction of *c-fos*, *Arc* and *Egr2* mRNAs in the mouse brain (Figs. 3b and 4). This profile resembles the TFPs by haloperidol, aripiprazole, and olanzapine in the basal ganglion. It is because MAP can induce *c-fos*, *Arc*, and *Egr2* via D1R in the striatonigral medium spiny neurons (MSNs) in the basal ganglia [49–51], while the antipsychotics can elicit these IEG changes mainly by inhibiting the D2R-mediated G_i/o_ signals in the striatopallidal MSNs [52]. In addition, MAP is reported to have both antipsychotic and propsychotic effects dependent on its concentration [53]. Psychostimulants such as MAP and amphetamine are clinically used to improve attention and cognition, and give rise to feeling of alertness, increased energy, and euphoria [54]. The *c-fos*, *Arc* and *Egr2* increases by MAP in basal ganglion as well as prefrontal cortex and hippocampus might be responsible for these effects.

The complex feature of MAP also appears to reflect conspicuous *Ccn1* transcript levels induced in the analyzed regions (Figs. 3b and 4). PCP and MAP have been reported to cause a prominent up-regulation of the neocortical expression of the secreted extracellular matrix-associated protein-encoded *Ccn1* gene in rat brain [17]. We observed that PCP and MK-801 elicit robust expression of *Ccn1* in the four brain regions, indicating that inhibition of NMDA signaling could induce robust *Ccn1* expression. This suggests that MAP also might inhibit NMDA signaling in some neurons of these regions, which could aggravate symptoms of schizophrenia. To demonstrate this hypothesis, further investigations are needed on a molecular basis.

In conclusion, we show that the distinctive temporal and spatial TFPs induced by antipsychotic or propsychotic drugs represent their pharmacological features. This assay system may provide the basis for rational design of signaling pathway-specific neuropharmacological drugs.

## Supporting Information

S1 Fig Schematic diagrams of brain regions in rodents selected for qRT-PCR analysis of changes in IEG mRNA expression. Prefrontal cortex and hippocampus in sagittal slice (left) and nucleus accumbens (NAc) and striatum in coronal slice (right), highlighted in pale orange, were analyzed in this study. Schematic was modified from the mouse brain atlas of Paxinos and Watson [55].
(TIF)

S1 Table Mouse TaqMan primer, probe and oligo template sequences (XLSX)

S2 Table Fold changes of time-course gene expression profiling by treatment of antipsychotic drugs. Fold changes in gene expression, normalized to *cyclophilin A* (*Ppia*) and relative to the expression of their corresponding vehicle group at the same time point, are calculated for each sample. Data are mean± SEM., n = 5: *p<0.05, **p<0.01, significantly different from vehicle in unpaired t-test with Welch’s correction. Antipsychotics; haloperidol (0.3 mg/kg, p.o.), aripiprazole (3 mg/kg, p.o.), olanzapine (10 mg/kg, p.o.) and clozapine (30 mg/kg, p.o.).
(XLSX)

S3 Table Fold changes of dose-response gene expression profiling by treatment of antipsychotic drugs. Fold changes in gene expression, normalized to *cyclophilin A* (*Ppia*) and relative to the expression of their corresponding vehicle group at a dose of 0 mg/kg, are calculated for each sample. Data are mean± SEM., n = 5: #p<0.025, significantly different from vehicle in Williams’ test. Antipsychotics; haloperidol (1 hr treatment, 0.03 mg/kg to 1 mg/kg, p.o.), aripiprazole (2 hrs treatment, 0.1 mg/kg to 3 mg/kg, p.o.), olanzapine (1 hr treatment, 0.3 mg/kg to 10 mg/kg, p.o.) and clozapine (1 hr treatment, 1 mg/kg to 30 mg/kg, p.o.).
(XLSX)

S4 Table Fold changes of time-course gene expression profiling by treatment of D2R antagonist/agonist. Fold changes in gene expression, normalized to *cyclophilin A* (*Ppia*) and relative to the expression of their corresponding vehicle group at the same time point, are calculated for each sample. Data are mean± SEM., n = 5: *p<0.05, **p<0.01, significantly different from vehicle in unpaired t-test with Welch’s correction. Selective D2R antagonist sulpiride (100 mg/kg, i.p.) and agonist quinpirole (10 mg/kg, i.p.).
(XLSX)

S5 Table Fold changes of time-course gene expression profiling by treatment of propsychotic drugs. Fold changes in gene expression, normalized to *cyclophilin A* (*Ppia*) and relative to the expression of their corresponding vehicle group at the same time point, are calculated for each sample. Data are mean± SEM., n = 5: *p<0.05, **p<0.01, significantly different from vehicle in unpaired t-test with Welch’s correction. Propsychotic agents; MAP (3 mg/kg, s.c.), PCP (10 mg/kg, s.c.) and MK-801 (1 mg/kg, s.c.).
(XLSX)

S6 Table Fold changes of dose-response gene expression profiling by treatment of propsychotic agents. Fold changes in gene expression, normalized to *cyclophilin A* (*Ppia*) and relative to the expression of their corresponding vehicle group at a dose of 0 mg/kg, are calculated for each sample. Data are mean± SEM., n = 5: #p<0.025, significantly different from vehicle in Williams’ test. Propsychotic agents; MAP (1 hr treatment, 1 mg/kg to 10 mg/kg, s.c.), PCP (1 hr treatment, 1 mg/kg to 10 mg/kg, s.c.), MK-801 (1 hr treatment, 0.1 mg/kg to 3 mg/kg, s.c.).
(XLSX)
