# Supplementary figures and images for: Temporal and Spatial Transcriptional Fingerprints by Antipsychotic or Propsychotic Drugs in Mouse Brain
Source: PLoS One. 2015 Feb 18;10(2):e0118510. doi: 10.1371/journal.pone.0118510 (PMC4334909; doi:10.1371/journal.pone.0118510)

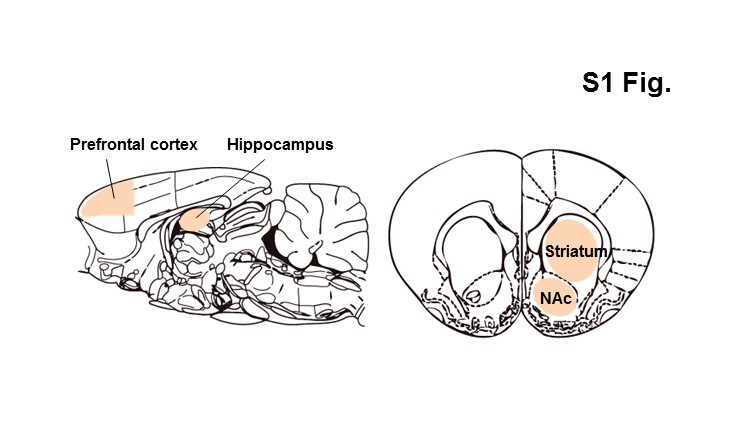

Supplement: S1 Fig — Prefrontal cortex and hippocampus in sagittal slice (left) and nucleus accumbens (NAc) and striatum in coronal slice (right), highlighted in pale orange, were analyzed in this study. Schematic was modified from the mouse brain atlas of Paxinos and Watson [55]. (TIF) [file pone.0118510.s001.tif]
